# Communicating the relative health risks of E-cigarettes: An online experimental study exploring the effects of a comparative health message versus the EU nicotine addiction warnings on smokers’ and non-smokers’ risk perceptions and behavioural intentions

**DOI:** 10.1016/j.addbeh.2019.106177

**Published:** 2020-02

**Authors:** Catherine Kimber, Daniel Frings, Sharon Cox, Ian P. Albery, Lynne Dawkins

**Affiliations:** Centre for Addictive Behaviours Research, Division of Psychology, School of Applied Sciences, 103 Borough Road, London South Bank University, London SE1 0AA, United Kingdom

**Keywords:** EC, E-cigarettes or Electronic cigarettes, EU-TPD, European Union Tobacco Products Directive, COMP, Comparative Health message (“*Use of this product is much less harmful than smoking*”), ONS, Office for National Statistics, MTSS, Motivation to Stop Scale, FTCD, Fagerström Test for Cigarette Dependence, Electronic cigarettes, Warning labels, Tobacco products directive, Health messages, Risk Perceptions, Quit intentions

## Abstract

**Introduction:**

This study investigated the effects of the European Union Tobacco Products Directive [EU-TPD] Article 20 E-cigarette (EC) health warnings (“*This product contains nicotine which is a highly addictive substance.* [*It is not recommended for non-smokers.*]”) and a comparative harm message (“*Use of this product is much less harmful than smoking”* [COMP]) on smokers’ and non-smokers’ perceptions and behavioural intentions.

**Methods:**

2495 UK residents (1283 smokers and 1212 non-smokers) self-reported perceived *harm*, *addictiveness*, *EC effectiveness*, *social acceptability,* and *intentions to purchase* and *use EC,* and in smokers, *intentions to quit* and *intentions to use EC in future quit attempts.* These were measured before and after exposure to EC images containing either the TPD, COMP, TPD + COMP or no message.

**Results:**

Non-smokers had higher harm, addictiveness and lower social acceptability perceptions. TPD presence increased, whilst COMP decreased, harm and addictiveness perceptions in both groups. For smokers only, harm perceptions were lower following exposure to COMP alone vs. no message. For non-smokers the TPD increased harm perceptions vs. no message. There were no effects on social acceptability, EC effectiveness or use intentions. In smokers only, purchase and quit intentions were higher following exposure to the COMP alone.

**Conclusion:**

TPD messages may be effective smoking prevention tools, although the COMP message was more effective in reducing harm perceptions and increasing use intentions in smokers. That COMP did not increase use intentions in non-smokers suggest that such exposures may potentially act as an effective harm reduction tool without resulting in increased uptake among non-smokers.

## 1 Introduction

Around 3.2 million adults in Great Britain currently use an EC (ASH, 2018). The most commonly cited reason for use is to reduce or quit smoking (ASH, 2018, ONS, 2018) and there is increasing evidence for their effectiveness for smoking cessation (Hajek et al., 2019, Jackson et al., 2019). E-cigarettes (EC) can be considered as tobacco harm reduction products as evidence suggests that they are considerably less harmful than tobacco cigarettes (Chen et al., 2017, Goniewicz et al., 2017, Goniewicz et al., 2014, Hecht et al., 2015, McAuley et al., 2012, Schripp et al., 2013, Shahab et al., 2017, Stephens, 2017). It is estimated that EC carry approximately 5% of the health risk associated with tobacco smoking (McNeill et al., 2015, McNeill et al., 2018, Royal College of Physicians & Group, 2016). Public health bodies and policy-makers (in the UK) recommend that smokers who are unwilling or unable to quit should be encouraged to switch to EC (McNeill et al., 2015, McNeill et al., 2018, Royal College of Physicians & Group, 2016). However, misperceptions of harm continue to increase. In Great Britain only 13% of survey respondents correctly believed that EC are considerably less harmful than tobacco smoking (ASH, 2017) while in a sample of 4058 Greek residents, 5% perceived EC to be less harmful than cigarettes (Farsalinos, Siakas, Poulas, Voudris, Merakou, & Barbouni, 2018). A recent longitudinal survey (N = 1720 UK current and former smokers) showed that just under half of respondents overestimated the harms of EC with 57.3% perceiving EC as less harmful than smoking (Wilson, Partos, McNeill, & Brose, 2019). These misperceptions were more prevalent in smokers who had never tried vaping suggesting that inaccurate information might be a contributing factor in deterring smokers from trying ECs (Wilson et al., 2019). To the extent that there is a need to redress these misperceptions, messages which communicate the relative risks of vaping compared to smoking may be of utility.

Warning labels, as used on tobacco products, have been the cornerstone of a comprehensive tobacco control plan aimed at both deterring non-smokers from trying the products whilst communicating the risks of smoking to smokers. How best to communicate risks of vaping (to deter non-smokers) while seeking to attract smokers to switch to a less harmful product, remains an ongoing question. Current messages on EC packaging present a standardised addiction health warning covering 30% of the pack. Article 20 of the EU Tobacco Products Directive [TPD] (European Union, 2014) requires that EC packets and refill products carry a health warning stating, either: i) ‘*This product contains nicotine which is a highly addictive substance*’, or ii) ‘*This product contains nicotine which is a highly addictive substance. It is not recommended for use by non-smokers*’. Given current misperceptions and concerns around addiction per se, warning messages which focus solely on the potential of EC to lead or maintain addiction may maintain public misperceptions of the harms of EC and discourage use amongst smokers. Indeed, concerns over substituting one addiction for another is the most commonly cited reason in deterring *never vaped* smokers from EC use (ASH, 2017).

To date, research exploring the impact of EC health warnings has been conducted outside of Europe, thus potential effects of the mandatory TPD nicotine addiction warning messages are underexplored. Experimental work has shown that exposure to EC advertisements (Berry, Burton, & Howlett, 2017) and EC health warnings conveying health and addiction risks, led to increased harm and addictiveness perceptions and decreased use intentions in smokers, EC users (Berry et al., 2017), and young non-smokers (Czoli et al., 2016, Mays et al., 2016). However, these effects are not equivocal (e.g. see Mays et al., 2016, Wackowski et al., 2019). Because current TPD messages focus on the absolute risks of nicotine use, they may actually deter use in smokers and undermine the potential of EC to assist a change in smoking behaviour. Whilst reducing appeal amongst non-smokers is clearly desirable, effective health messaging should communicate risks without discouraging smokers. Comparative health messages, which focus on the reduced risks of EC in relation to tobacco smoking, may provide a viable alternative and/or complement to the EU-TPD messages, and increase the perceived utility and value of using EC as a quit aid.

### 1.1 Aims

This study aimed to investigate the effects of the EU-TPD EC health warnings and a comparative harm message (COMP) on smokers’ and non-smokers’ perceptions of harm, addictiveness, effectiveness and social acceptability of EC. We also aimed to explore the effects of the EU-TPD warnings on smokers’ and non-smokers’ intentions to purchase and use an EC, and on smokers’ quit intentions and intentions to use EC in future quit attempts. Lastly, the potential effects of providing a comparative harm message either in addition to the TPD warning or as a stand-alone message were also explored.

## 2 Method

### 2.1 Design

Ethical approval was granted by the School of Applied Sciences Ethics Committee at London South Bank University (4/07/2018; reference SAS1815). Data were collected in the UK between December 2018 and January 2019. A 2 × 2 × 2 × 2 factorial design with Smoking status (Smoker vs. Non-smoker), TPD presence (Presence vs. Absence) and COMP presence (Presence vs. Absence) as between-subject factors, and Time (pre-post exposure of images/health messages) as a within-subjects factor. Dependent variables comprised self-reported perceived harm, addictiveness, social acceptability, effectiveness, intentions to purchase and use of EC, intentions to quit and intentions to use ECs in future quit attempts.

### 2.2 Participants

2495 UK residents were recruited (N = 1283 smokers; N = 1212 non-smokers; see Kimber, Frings, Cox, Albery, & Dawkins, 2018 for sample size calculation). Participants were enrolled via a recruitment panel agency MRFGR (Market Research Focus Group Recruiter). Remuneration was on a point-based system, each participant received 6 points (a minimum of 150 points can be redeemed via PenPal, Amazon gift cards, lottery tickets and charity donations). The sample was matched to the target population of smokers’ demographics in the general population using ONS data concerning age, gender and socio-economic status (SES). The same sampling stratification was applied to non-smokers. Inclusion criteria were: adults aged 18+, resident in the UK, and fluent in English. Exclusion criteria were: under 18 years of age, resident outside of the UK, exclusive vapers, dual users (concurrent use of EC and any tobacco product).

### 2.3 Materials and measures

#### 2.3.1 Measures

All measures were completed online. Demographic variables included gender, age, ethnicity, occupation, and highest attained qualification in line with data collected by the Office for National Statistics (ONS) and others (e.g. Windsor-Shellard, Pullin, & Horton, 2018).

Participants were classified as ‘non-smoker’, ‘daily smoker’ and ‘occasional smoker’ (see Kimber et al., 2018 for full definitions). As per our published protocol (Kimber et al., 2018), daily and occasional smokers were combined so analyses included a binary measure of smoking status (Smoker vs. non-smoker).

For smokers, motivation to quit was measured using the Motivation to Stop Scale ([MTSS] (Kotz, Brown, & West, 2013)). Cigarette dependence was measured using the Fagerström Test for Cigarette Dependence (FTCD) (Fagerström, 2012).

The COMP message was generated in a pilot study (which gathered ratings on how clear, understandable and believable a range of relative risk health messages were) and selected for its higher than average overall mean rating and its brevity (see Kimber et al., 2018 for further details on the conceptualisation of the message). Each message was placed on a series of 4 different types of EC packs (see Fig. 1 in supplementary materials) for a standardised period of 30 s. In line with the current EU-TPD requirements all messages occupied 30% of the surface of the pack and were presented in black Helvetica bold type on a white background.

Prior to, and following the experimental exposure to one of the EC health messages (see Table 1), perceptions of EC and intentions were measured on the following scales: i) harmfulness, ii) addictiveness and ii) socially acceptable, iv) effectiveness as a cessation aid, v) intention to use, vi) intention to purchase and, for smokers only, vii) intentions to quit and use e-cigarettes in a future quit attempt (all on a 7-point rating scales anchored at “Extremely”, to “Not at all”; see Kimber et al., 2018 for the full list of constructs). To minimise response bias, unrelated questions (e.g. “*Which e-cigarette did you think looked most like a cigarette?*”) were presented following exposure of the message stimuli in addition to outcome measures.

**Table 1:** Stimuli parameters and statements used in each condition.

Conditions	Parameters	Message statements
TPD1	TPD health warning as per currently implemented in the UK	“*This product contains nicotine which is a highly addictive substance*”
TPD2	TPD longer health warning as currently implemented in many EU countries	“*This product contains nicotine which is a highly addictive substance. It is not recommended for non-smokers*”
COMP	Same parameters used for the TPD warning labels; font, font colour, size and placement on the pack	“*Use of this product is much less harmful than smoking*”
TPD1+	The TPD health warning (TPD1) in combination with the comparative message (using the same parameters above)	“*This product contains nicotine which is a highly addictive substance. Use of this product is much less harmful than smoking”*
TPD2+	The TPD longer health warning (TPD2) in combination with the comparative message (using the same parameters as above)	*“This product contains nicotine which is a highly addictive substance. It is not recommended for non-smokers. Use of this product is much less harmful than smoking”*
*No message*	A *no message* condition using the same EC pack images	

#### 2.3.2 Procedure

The full procedure is outlined in our protocol paper (Kimber et al., 2018). Upon providing online consent, demographic data were collected and followed by the FTCD and MTSS questionnaires (smokers only). All participants were then asked about their perceived harm, addictiveness, social acceptability, effectiveness, intentions to purchase and use of EC. Smokers were also asked about their intentions to quit and intentions to use ECs in future quit attempts. Each participant was randomised to one of the six messages on EC pack images (see Table 1) and viewed sequentially; four EC packs containing one of the messages (or no message for the control group) each for 30 s. Following exposure of the stimuli, filler questions and outcome measures were completed. Finally, participants were debriefed.

#### 2.3.3 Data analysis

As per our published protocol, a series of ANCOVAs were conducted with 3 between-factors: Smoking Status (Smoker vs. Non-smoker), TPD Presence (Present vs. Absent), COMP Presence (Present vs. Absent) with DVs consisting of Time 2 perceived ratings of EC i) harm, ii) addictiveness, iii) social acceptability, iv) effectiveness, v) intention to quit, vi) intention to purchase and vii) intention to use ECs to observe the main effects and interactions whilst controlling for Time 1 measurements of the DV. All main and interactive simple effects were tested, with planned a-priori comparisons between the TPD alone condition against i) TPD + COMP, ii) no TPD/no COMP iii) no TPD/COMP, and the COMP alone condition against i) TPD + COMP, ii) no TPD/no COMP, iii) TPD/no COMP. These were conducted at each level of smoking status. As per protocol, for secondary analyses, the same ANCOVAs were repeated i) controlling for cigarette dependence, previous e-cigarette exposure, and baseline intentions to quit and, ii) exploring the interactions with demographic variables (age, sex and occupation). For i) only significant main effects and interactions are reported here and for ii) adding demographics co-variates did not change the overall pattern of main effects and interactions reported here; these results will be reported separately and made available via the Open Science Framework: osf.io/ta4vx. As per our published protocol, outliers were calculated (±2.5 SD) for each variable before removal, the total numbers discarded differed per variable and ranged between 15 and 69. When outliers were included the effects of the COMP vs. no message on smokers’ harm perceptions and the effect of the TPD vs. no message on smokers’ perceptions of EC addictiveness fell short of statistical significance. In addition, a further effect of COMP vs TPD was found. Smokers’ intentions to use an EC in a quit attempt next month were higher after exposure to the COMP vs. the TPD.

## 3 Results

### 3.1 Demographics

Table 2 summarises demographics and smoking characteristics. No differences were observed between conditions on key demographic characteristics (*p*s > 0.05; See supplementary materials for exact test statistics and p values).

**Table 2:** Sample characteristics (N = 2495).

	N	%	Mean	SD	Min	Max
Sex	**–**	**–**	**–**	**–**	**–**	**–**
Male	1173	47	**–**	**–**	**–**	**–**
Female	1322	53	**–**	**–**	**–**	**–**
Ethnicity			**–**	**–**	**–**	**–**
White	2303	92.3	**–**	**–**	**–**	**–**
Black/African/Caribbean	38	1.5	**–**	**–**	**–**	**–**
Mixed/Multiple ethnic background	44	1.8	**–**	**–**	**–**	**–**
South Asian/Indian/Pakistani/Bang	75	3.0	**–**	**–**	**–**	**–**
Chinese/Other Asian background	21	0.8	**–**	**–**	**–**	**–**
Other	14	0.5	**–**	**–**	**–**	**–**
Occupation			**–**	**–**	**–**	**–**
Routine and manual	723	29.0	**–**	**–**	**–**	**–**
Intermediate	477	19.1	**–**	**–**	**–**	**–**
Managerial & professional	684	27.4	**–**	**–**	**–**	**–**
Never worked & Long term unemployed	611	24.5	**–**	**–**	**–**	**–**
Highest qualification to date			**–**	**–**	**–**	**–**
Degree (or equivalent)	729	29.2	**–**	**–**	**–**	**–**
Higher education (below degree level)	259	10.4	**–**	**–**	**–**	**–**
A-levels or Highers	445	17.8	**–**	**–**	**–**	**–**
ONC or National level BTEC	151	6.1	**–**	**–**	**–**	**–**
O-Level or GCSE equivalent (A-C)	277	16.5	**–**	**–**	**–**	**–**
GCSE (D-E), CSE (2–5) or standard grade (4–6)		11.1	**–**	**–**	**–**	**–**
Other qualifications	100	4.0	**–**	**–**	**–**	**–**
No formal qualifications	123	4.9	**–**	**–**	**–**	**–**
Age segments	**–**	**–**	**–**	**–**	**–**	**–**
18–49	1398	56	**–**	**–**	**–**	**–**
50+	1097	44	**–**	**–**	**–**	**–**
Smoking status			**–**	**–**	**–**	**–**
Daily smokers	1158	44.41	**–**	**–**	**–**	**–**
Occasional smokers	125	5.01	**–**	**–**	**–**	**–**
Non-smoker	1212	48.6	**–**	**–**	**–**	**–**
Past EC use			**–**	**–**	**–**	**–**
Never used	1768	70.9	**–**	**–**	**–**	**–**
Past experimentation^1^	301	12.1	**–**	**–**	**–**	**–**
Former occasional users^2^	253	10.1	**–**	**–**	**–**	**–**
Former daily users	173	6.9	**–**	**–**	**–**	**–**
Quit attempts (Smokers N = 1283)						
Yes	861	67.11	**–**	**–**	**–**	**–**
No	422	32.89	**–**	**–**	**–**	**–**
Number of past quit attempts (N = 1283)	**–**	**–**	3.87	5.37	1	100
Number of years smoking			26.23	14.95	0.8	64
*FTCD*^3^ (N = 1283)	**–**	**–**	4.08	2.48	0	10
*MTSS*^4^ (N = 1283)	**–**	**–**	3.06	1.69	1	7

Note.

1 Past experimentation = Used an EC very rarely in the past and no longer use it.

2 Former occasional users = Used an EC occasionally (not daily) in the past and no longer use it.

3 FTCD = Fagerström Test for Cigarette Dependence.

4 MTSS = Motivation to Stop [Smoking] Scale; FTCD and MTSS both were measured in smokers only.

TPD1 and 2 were combined in the analyses as scores of risk perceptions and intentions did not differ (ps > 0.05), thus subsequent references to TPD are to both TPD messages combined. All confidences intervals reported are at 95%.

#### 3.1.1 Perceptions of EC related to harms

ANCOVAs revealed a main effect of Smoking Status F(1, 2408) = 63.21, p < .001, ƞ_p_^2^ = 0.03; Non-smokers perceived EC as more harmful compared to smokers (Smokers, M = 4.97 [CI: 4.91–5.01] vs. Non-smokers M = 5.28 [CI: 5.22–5.33]). There was also a main effect of TPD Presence F(1, 2408) = 31.89, p < .001, ƞ_p_^2^ = 0.01. Harm-related perceptions were greater in those exposed to TPD (M = 5.23 [CI: 5.19–5.27]) compared to TPD absence conditions (M = 5.01 [CI: 4.95–5.07]). After adjusting for Motivation to Quit and Cigarette Dependence, a main effect of COMP was shown F(1, 1238) = 7.11, p = .008, ƞ_p_^2^ = 0.006. Following exposure of the COMP message, smokers perceived EC as less harmful compared to exposure to other messages. A-priori comparisons revealed that both smokers and non-smokers exposed to the TPD perceived EC as significantly more harmful compared to those exposed to the COMP alone (see Table 3). In non-smokers only, exposure to the TPD alone showed increased harm perceptions compared to *No message*. In both smokers and non-smokers, significantly lower scores were shown following exposure to the COMP alone message compared to the TPD alone and the TPD + COMP combined. However, in smokers only, exposure to the COMP alone led to reduced harm perceptions compared to *No message* (see Table 3). A significant Smoking status by COMP presence interaction was shown, F(1, 2408) = 6.325, p = .012, ƞ_p_^2^ = 0.003; Simple effects revealed that smokers’ perceptions differed significantly when COMP was present (M = 4.89 [CI: 4.81–4.92]) compared to when it was absent (M = 5. 05 [CI: 4.97–5.12]). Non-smokers’ harm perceptions were higher than that of smokers; when COMP was present (M = 5.29 [CI: 5.21–5.37]) harm perceptions were slightly higher compared to when COMP was absent (M = 5.26 [CI: 5.18–5.33]).

**Table 3:** Means [95% CI] perceptions related to EC per smoking status with TPD alone vs. COMP alone vs. TPD + COMP vs. *No message*.

	Smokers				Non-smokers			
	N	M [95%CI]	A-priori	p	N	M [95%CI]	A-priori	p
**Perceptions**								
**Harm**	1245		1 vs. 2	0.085	1172		1 vs. 2	0.353
1. TPD alone		4.89 [4.80–4.98]	1 vs. 3	0.096		5.66 [5.59–5.74]	1 vs. 3*	0.001
2. TPD + COMP		4.78 [4.69–4.87]	1 vs. 4*	0.001		5.61 [5.54–5.69]	1 vs. 4*	0.002
3. *No message*		4.76 [4.63–4.89]	2 vs. 4*	0.007		5.33 [5.22–5.44]	2 vs. 4*	0.019
4. COMP alone		4.55 [4.41–4.69]	3 vs. 4*	0.034		5.45 [5.33–5.56]	3 vs. 4	0.150
**Addictiveness**	1238		1 vs. 2	0.109	1171		1 vs. 2	0.254
1. TPD alone		5.18 [5.11–5.16]	1 vs. 3*	0.012		6.02 [5.94–6.09]	1 vs. 3*	0.001
2. TPD + COMP		5.28 [5.20–5.36]	1 vs. 4*	0.007		5.96 [5.88–6.03]	1 vs. 4*	0.001
3. *No message*		5.00 [4.89–5.12]	2 vs. 4*	0.001		5.64 [5.54–5.75]	2 vs. 4*	0.001
4. COMP alone		4.98 [5.86–5.11]	3 vs. 4	0.787		5.66 [5.55–5.77]	3 vs.4	0.834
**Effectiveness**	1268		1 vs. 2	0.376	1212		1 vs. 2	0.866
1. TPD alone		4.36 [4.26–4.46]	1 vs. 3	0.335		4.14 [4.04–4.23]	1 vs. 3	0.387
2. TPD + COMP		4.43 [4.33–4.53]	1 vs. 4	0.412		4.12 [4.03–4.22]	1 vs. 4	0.343
3. *No message*		4.28 [4.13–4.42]	2 vs. 4	0.898		4.21 [4.08–4.34]	2 vs. 4	0.278
4. COMP alone		4.44 [4.29–4.59]	3 vs. 4	0.128		4.22 [4.08–4.36]	3 vs. 4	0.922
**Social acceptability**	1244		1 vs. 2	0.892	1212		1 vs. 2	0.621
1. TPD alone		4.75 [4.67–4.84]	1 vs. 3	0.579		3.87 [3.77–3.96]	1 vs. 3	0.847
2. TPD + COMP		4.75 [4.66–4.83]	1 vs. 4	0.343		3.83 [3.74–3.92]	1 vs. 4	0.690
3. *No message*		4.71 [4.59–4.84]	2 vs. 4	0.399		3.85 [3.72–3.98]	2 vs. 4	0.989
4. COMP alone		4.68 [4.55–4.81]	3 vs. 4	0.727		3.83 [3.69–3.97]	3 vs. 4	0.854

Note.

* Significant differences were accepted at level p ≤ 0.05 using Univariate a-priori comparisons and estimated marginal means with 95% confidence intervals; A-priori comparisons are: Level 1 vs. 2 = TPD alone vs. TPD + COMP; Level 1 vs. 3 = TPD alone vs. *No message*; Level 1 vs. 4 = TPD alone vs. COMP alone; Level 2 vs. 4 = TPD + COMP vs. COMP alone; Level 3 vs. 4 = *No message* vs. COMP alone.

#### 3.1.2 Perceptions of EC related to addictiveness

There was a main effect of Smoking Status F(1, 2400) = 68.55, p < .001, ƞ_p_^2^ = 0.03; Smokers perceived EC as less addictive than non-smokers (Smokers, M = 5.31 [CI: 5.26–5.36] vs. non-smokers M = 5.61 [CI: 5.56–5.66]). There was a main effect of TPD presence F(1, 2400) = 65.31, p < .001, ƞ_p_^2^ = 0.03; significantly increased scores were shown for those who had been exposed to the TPD compared to no presence conditions (TPD present M = 5.60 [CI: 5.56–5.64] vs. TPD absent M = 5.32 [CI: 4.95–5.07]). A-priori comparisons revealed that compared to the COMP alone and *No message* conditions, exposure to the TPD increased perceptions of addictiveness. In addition, exposure to the COMP alone compared to both the TPD and the COMP combined (TPD + COMP) led to significantly lower perceptions of addictiveness in both smokers and non-smokers (see Table 3).

#### 3.1.3 Perceptions of EC related to their effectiveness

No significant main effects, interaction nor a-priori contrasts were identified (*p*s > 0.05) (see Table 3).

#### 3.1.4 Perceptions of social acceptability of EC

There was a main effect of Smoking Status, F(1, 2447) = 11.01, p < .001, ƞ_p_^2^ = 0.004; Smokers (M = 4.36 [CI: 4.30–4.41]) perceived EC to be more socially acceptable compared to non-smokers (M = 4.22 [CI: 4.16–4.28]). All remaining main effect, interaction and a-priori comparisons were not significant (ps > 0.05) (see Table 3).

#### 3.1.5 Intentions to purchase an EC next month and in 6 months

In the next month and in 6 months respectively, ANCOVAs revealed a main effect of Smoking Status F(1, 2416) = 42.46, p < .001, ƞ_p_^2^ = 0.02 and F(1, 2417) = 55.47, p < .001, ƞ_p_^2^ = 0.02. Smokers reported higher intentions to purchase an EC compared to non-smokers (Smokers M = 1.93 [CI: 1.88–1.98] vs. non-smokers M = 1.66 [CI: 1.60–1.72]) and in the next 6 months respectively (Smokers M = 2.04 [CI: 1.98–2.09] vs. non-smokers M = 1.73 [CI: 1.67–1.79]). A-priori comparisons revealed higher purchase intentions in those exposed to the COMP alone compared to the TPD alone in smokers only (see Table 4). All remaining main and interaction effects were not significant (ps > 0.05).

**Table 4:** Means [95% CI] Intentions per smoking status with TPD alone vs. COMP alone vs. TPD + COMP vs. *No message*.

	Smokers				Non-smokers			
	N	M [95%CI]	A-priori	p	N	M [95%CI]	A-priori	p
**Intentions**								
**EC Purchase next month**	1283		1 vs. 2	0.600	1133		1 vs. 2	0.885
1. TPD alone		2.44 [2.33–2.55]	1 vs. 3	0.840		1.01 [1.00–1.02]	1 vs. 3	0.927
2. TPD + COMP		2.48 [2.37–2.59]	1 vs. 4*	0.049		1.01 [1.00–1.02]	1 vs. 4	0.492
3. *No message*		2.46 [2.30–2.62]	2 vs. 4	0.119		1.01 [1.00–1.02]	2 vs. 4	0.562
4. COMP alone		2.64 [2.47–2.80]	3 vs. 4	0.126		1.00 [0.99–1.02]	3 vs. 4	0.500
**EC Purchase in 6 months**	1283		1 vs. 2	0.445	1134		1 vs. 2	0.493
1. TPD alone		2.63 [2.53–2.74]	1 vs. 3	0.610		1.01 [1.01–1.02]	1 vs. 3	0.822
2. TPD + COMP		2.69 [2.58–2.80]	1 vs. 4	0.582		1.01 [1.00–1.02]	1 vs. 4	0.378
3. *No message*		2.68 [2.53–2.83]	2 vs. 4	0.965		1.01 [1.00–1.02]	2 vs. 4	0.722
4. COMP alone		2.68 [2.53–2.85]	3 vs. 4	0.962		1.01 [0.99–1.02]	3 vs. 4	0.564
**EC Use next month**	1283		1 vs. 2	0.500	1130		1 vs. 2	0.655
1. TPD alone		2.52 [2.41–2.62]	1 vs. 3	0.973		1.01 [1.00–1.02]	1 vs. 3	0.111
2. TPD + COMP		2.57 [2.46–2.68]	1 vs. 4	0.144		1.01 [1.00–1.01]	1 vs. 4	0.696
3. *No message*		2.52 [2.37–2.68]	2 vs. 4	0.350		1.02 [1.01–1.03]	2 vs. 4	0.964
4. COMP alone		2.66 [2.50–2.82]	3 vs. 4	0.218		1.00 [0.99–1.02]	3 vs. 4	0.092
**EC Use in 6 months**	1283		1 vs. 2	0.843	1130		1 vs. 2	0.416
1. TPD alone		2.72 [2.61–2.82]	1 vs. 3	0.787		1.01 [1.00–1.02]	1 vs. 3	0.699
2. TPD + COMP		2.73 [2.62–2.84]	1 vs. 4	0.222		1.01 [1.00–1.02]	1 vs. 4	0.864
3. *No message*		2.74 [2.59–2.90]	2 vs. 4	0.286		1.02 [1.00–1.03]	2 vs. 4	0.419
4. COMP alone		2.84 [2.68–3.00]	3 vs. 4	0.407		1.01 [1.00–1.03]	3 vs. 4	0.858

Note. All intentions were measured on a 7-point rating scales with the anchors “Extremely likely to Not at all likely to” scoring from 7 to 1.

* Significant differences were accepted at level p ≤ 0.05 using Univariate a-priori comparisons and estimated marginal means with 95% confidence intervals; A-priori comparisons are Level 1 vs. 2 = TPD alone vs. TPD + COMP; Level 1 vs. 3 = TPD alone vs. *No message*; Level 1 vs. 4 = TPD alone vs. COMP alone; Level 2 vs. 4 = TPD + COMP vs. COMP alone; Level 3 vs. 4 = *No message* vs. COMP alone.

#### 3.1.6 Intentions to use an EC next month and in 6 months

A main effect of smoking status was shown only after adjusting for EC past exposure, F(1, 2403) = 42.06, p < .001, ƞ_p_^2^ = 0.02. Smokers reported higher intentions to use an EC compared to non-smokers (Smokers M = 1.97 [CI: 1.92–2.02] vs. non-smokers M = 1.70 [CI: 1.63–1.75]). All remaining main and interaction effects were not significant (ps > 0.05).

#### 3.1.7 Smokers’ intentions to quit and use EC in future quit attempts

For intentions to quit in the next month, ANCOVAs revealed a main effect of TPD Presence F(1,1278) = 5.26, p = .022, ƞ_p_^2^ = 0.004; When present, smokers’ intentions to quit were lower (M = 2.63 [CI: 2.57–2.70]) compared to when TPD was absent (M = 2.77 [CI: 2.67–2.87]). There was no main effect of COMP presence (COMP present M = 2.74 [CI: 2.65–2.82] vs. COMP absent M = 2.67 [CI: 2.58–2.75]; p = .248). There was no significant interaction (p = .201). A-priori comparisons revealed that smokers reported increased intentions to quit in the next month when showed the COMP alone. The COMP message significantly differed from exposure to the TPD and exposure to both messages combined (TPD + COMP) (Table 5).

**Table 5:** Means [CI] Smokers’ intentions with TPD alone vs. COMP alone vs. TPD + COMP vs. *No message*.

	N	M [95%CI]	A-priori	p
**Intentions (Smokers only)**				
**Quit next month**	1283		1 vs. 2	0.911
1. TPD alone		2.64 [2.54–2.73]	1 vs. 3	0.467
2. TPD + COMP		2.63 [2.53–2.72]	1 vs. 4*	0.016
3. *No message*		2.70 [2.56–2.84]	2 vs. 4*	0.013
4. COMP alone		2.85 [2.70–3.00]	3 vs. 4	0.142
**Quit in 6 months**	1283		1 vs. 2	0.862
1. TPD alone		3.13 [3.04–3.23]	1 vs. 3	0.989
2. TPD + COMP		3.12 [3.03–3.22]	1 vs. 4	0.120
3. *No message*		3.13 [3.00–3.27]	2 vs. 4	0.091
4. COMP alone		3.27 [3.13–3.41]	3 vs. 4	0.175
**EC Use in quit attempt next month**	732		1 vs. 2	0.317
1. TPD alone		1.16 [1.06–1.25]	1 vs. 3	0.867
2. TPD + COMP		1.22 [1.13–1.31]	1 vs. 4	0.216
3. *No message*		1.17 [1.04–1.30]	2 vs. 4	0.639
4. COMP alone		1.26 [1.12–1.40]	3 vs. 4	0.343
**EC Use in quit attempt in 6 months**	635		1 vs. 2	0.294
1. TPD alone		1.18 [1.08–1.29]	1 vs. 3	0.978
2. TPD + COMP		1.26 [1.16–1.36]	1 vs. 4	0.312
3. *No message*		1.18 [1.04–1.33]	2 vs. 4	0.840
4. COMP alone		1.28 [1.12–1.44]	3 vs. 4	0.385

Note. All intentions were measured on a 7-point rating scales with the anchors “Extremely likely to Not at all likely” scoring from 7 to 1; * Significant differences were accepted at level p ≤ 0.05 using a-priori comparisons and estimated marginal means with 95% confidence intervals; A-priori comparisons are: Level 1 vs. 2 = TPD alone vs. TPD + COMP; Level 1 vs. 3 = TPD alone vs. *No message*; Level 1 vs. 4 = TPD alone vs. COMP alone; Level 2 vs. 4 = TPD + COMP vs. COMP alone; Level 3 vs. 4 = *No message* vs. COMP alone.

For intentions to quit within 6 months, and intentions to use an EC in a quit attempt in the next month and in the next 6 months, no significant differences between conditions was shown (ps < 0.05).

## 4 Discussion

This study compared the effects of the EU-TPD EC nicotine addiction health warning to a comparative harm (COMP) message on smokers’ and non-smokers’ perceptions of EC. We also examined the effects of these messages on purchase intentions, and on smokers’ intentions to quit and to use an EC in a quit attempt. The potential effects of a comparative risk message either in addition to the TPD warnings or as a stand-alone message were also explored.

Smokers perceived EC as less harmful, less addictive and more socially acceptable than non-smokers. EC were perceived as more harmful following exposure to the TPD messages by both smokers and non-smokers. Following exposure to the COMP alone EC were perceived as less harmful by both smokers and non-smokers when compared to both messages combined. However, when compared with no message, reduction in perceptions of EC harm was shown after exposure to the COMP alone only in smokers. This suggests that adding the COMP as a stand-alone message to EC packs, may reduce smokers’ harm perceptions of EC whilst leaving non-smokers’ EC harm perceptions unaffected. For perceptions of EC addictiveness, the TPD and the COMP alone conditions differed significantly. Perceptions of EC addictiveness increased in both smokers and non-smokers following exposure to the TPD relative to those exposed to no messages and those exposed to the COMP. Similarly, smokers and non-smokers exposed to the COMP alone perceived EC as less addictive compared to those exposed to both the TPD and COMP messages combined. For EC purchase intentions smokers reported increased intentions to buy in the next month (although not in the next 6 months) compared to non-smokers. Critically, in smokers (not non-smokers), exposure to the COMP message increased intentions to purchase an EC in the next month compared to exposure to the TPD messages; this was the case only when the COMP message was presented alone. The COMP message led to higher quit intentions compared to the TPD in smokers only.

That non-smokers were more likely to endorse beliefs about EC addictiveness and harm compared to smokers corroborates previous evidence (Mays et al., 2019, O’Brien et al., 2017, Wilson et al., 2019). It is unsurprising that non-smokers were more likely to be influenced by a health warning such as the TPD because it is likely to align with their pre-existing tobacco and nicotine beliefs.

The finding that perceptions of harm differed between smokers and non-smokers following exposure to the COMP message alone compared to no messages suggests that smokers may be more receptive to the COMP than non-smokers. This is encouraging to the extent that reduced harm messages which convey relative risks and encourage a switch away from smoking is important for smokers, but at the same time should not encourage use among non-smokers. In other words, it suggests specificity for better targeting these individuals by increasing engagement about the relative risks of EC. However, when comparing the TPD messages to no message, the increase in harm perceptions was only present for non-smokers. To some extent, this demonstrates the effectiveness of the TPD messages as a smoking prevention tool without also increasing harm perceptions among smokers.

Exposure to the COMP alone was also associated with reduced scores on perceptions of addictiveness of EC compared to the TPD and the TPD + COMP. Thus, COMP may act as a stand-alone message as opposed to as an adjunct to the TPD. Previous work which paired relative risks health statements with addiction warnings has failed to show any influence on addictiveness perceptions and use intentions (Wackowski et al., 2019). Given this study also found a reduction in credibility and believability of the message (Wackowski et al., 2019), one possible explanation for this loss in credibility could be attributed to the pairing of a reduced risk statement and a warning label (warning against harm) which is likely to be perceived as conflicting and ambiguous, leading to message rejection (Katz, Erkkinen, Lindgren, & Hatsukami, 2018). Another likely factor to have influenced our findings could be that the warning message preceding the reduced risk (TPD + COMP), it is unclear whether a different ordering would have yielded different outcomes. An alternative explanation for the TPD to exert such influence even when accompanied by the COMP is that perceptions may have been influenced by increasing familiarity (via past repeated exposure to the TPD). Moreover, because public perceptions of nicotine’s addictiveness and harmfulness are likely to be an established belief set available for processing (e.g. ASH, 2017, East et al., 2018, Majeed et al., 2017, Riahi et al., 2019, Wilson et al., 2019), it is likely that individuals will bring such grounded beliefs when interacting with any message. Thus, participants may be processing messages based on more belief-based heuristics.

The TPDs and the COMP messages did not differ in their influence on perceptions of EC as effective stop smoking aids nor did they affect perceptions on social acceptability, perhaps because none focus on effectiveness or social acceptability. Because such a focus may influence switching amongst smokers, they may require separate attention from messages focusing on harm.

As expected, the COMP led to higher purchase intentions compared to the TPD in smokers only, but these effects were lost when participants were asked to rate their intentions over 6 months. There were no effects or differences in intentions to use EC in the future and overall ratings were low. Although previous studies (Berry et al., 2017, Cox et al., 2018, Lee et al., 2018, Mays et al., 2016), found that the TPD have the potential to deter smokers from substituting their tobacco for EC use, here, exposure to the TPD only, did not reduce smokers’ use intentions compared to no message. Thus, while COMP versus TPD may be more favourable towards encouraging harm reduction for smokers, TPD only versus no TPD may not necessarily discourage harm reduction for smokers. Exposure to the COMP alone led to greater quit intentions compared to the TPD and both messages combined (TPD + COMP). Thus, in this study, we demonstrate that presenting a message that conveys the relative risks of EC to smoking, can increase EC purchase and quit intentions in smokers.

Ideally, a comparative harm message would reduce perceptions of harm in both smokers and non-smokers, while increasing intentions to use for smokers. However, we found that although the COMP showed lower harm and addictiveness perceptions of EC in both smokers and non-smokers, the COMP alone against no message did not affect non-smokers’ perceptions whilst increasing purchase intentions in smokers only. Furthermore, the null effects of COMP on use intentions in non-smokers is a promising finding in that it suggests such exposure might not result in unintended effects.

### 4.1 Limitations

The study has several limitations. Although the sample was matched to the ONS data to represent the smoking population, it was self-selecting, potentially introducing response bias. Secondly, whilst findings of this study are informative and help further our understanding of communication of risks related to EC, these are confined to intentions. Prospective studies could focus on exploring how to translate intentions into behaviours.

## 5 Conclusions

The findings suggest that messages such as the COMP can help redress harm perceptions associated with EC and encourage smokers to switch to EC. When communicating health risks, it is important that messages are clear and unambiguous. Any future communication strategy to include a comparative message, such as the one developed here, has greater potential to impact as a stand-alone message.

## Role of funding sources

This study was funded by Cancer Research UK's Tobacco Advisory Group (CRUK Grant Number: 25855). The funder played no role in the design of the study and drafting of the manuscript.

## Authors’ contributions

LD and DF are joint lead principal researchers and grant holders for this project. Along with SC and IA, LD and DF conceived the original idea for the project, designed the study, refined the methodology and contributed to the grant application. CK led on the drafting of the manuscript and was responsible for the day-to-day running of the project and data collection. All authors contributed significantly to and edited drafts of this manuscript. All authors have read and approved the final manuscript.

## Declaration of Competing Interest

CK has no conflict of interest to declare.

DF is principal investigator on a randomised controlled trial funded by Allen Carr’s Easyway Ltd (ISRCN number: ISRCTN23584477). This trial is comparing the Allen Carr Easyway stop-smoking method to local NHS 1-1 stop smoking counselling service. The trial is being conducted independently, the protocol and analysis plan are both pre-registered and the research team are contractually free to independently publish the results of the trial regardless of the study outcome. DF has no other conflicts of interest to declare.

SC has provided consultancy services to UK life insurers on smoking cessation and reduce risk products prevalence rates.

IA is an investigator on a randomised controlled trial funded by Allen Carr’s Easyway Ltd (ISRCN number: ISRCTN23584477). This trial is comparing the Allen Carr Easyway stop-smoking method to local NHS 1-1 stop smoking counselling service. The trial is being conducted independently, the protocol and analysis plan are both pre-registered and the research team are contractually free to independently publish the results of the trial regardless of the study outcome.

LD has provided consultancy for the pharmaceutical industry (2015, 2017) and acted as an expert witness for an EC patent infringement case (2015). Between 2011 and 2013 she conducted research for several independent electronic cigarette companies for which the University of East London received funds. The EC companies involved had no input into the design, conduct or write up of these projects.
