# Supplementary material for: Communicating the relative health risks of E-cigarettes: An online experimental study exploring the effects of a comparative health message versus the EU nicotine addiction warnings on smokers’ and non-smokers’ risk perceptions and behavioural intentions
Source: Addict Behav. 2020 Feb;101:106177. doi: 10.1016/j.addbeh.2019.106177 (PMC6891257; doi:10.1016/j.addbeh.2019.106177)
Supplement: Supplementary data 1 [file mmc1.pdf]

Supplementary Materials:

Title: Communicating the Relative Health Risks of E-Cigarettes: An online experimental study exploring the Effects of a Comparative Health Message versus the EU Nicotine Addiction Warnings on Smokers' and Non-Smokers' Risk Perceptions and Behavioural Intentions.

Stimuli used in randomised conditions:

Participants were randomised to one of the six conditions (each condition consisted of 4 EC packs). Each message was placed on a series of 4 different types of EC packs (Figure 1) and each pack was displayed in a sequential order for a standardised period of 30 seconds. Note, images presented below act as a template, parameters were later set to comply with the current EU-TPD requirements (i.e. all messages occupied 30% of the surface of the pack and placed at the bottom of the pack; these were presented in black Helvetica bold type on a white background).

TPD1 CONDITION

E-cigarette #1

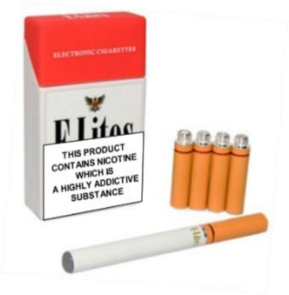

E-cigarette #2

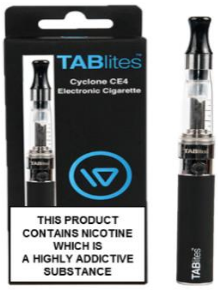

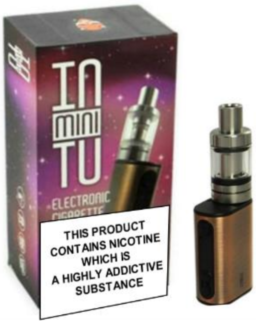

E-cigarette #3

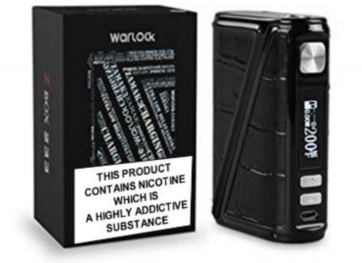

E-cigarette #4

TPD2 CONDITION

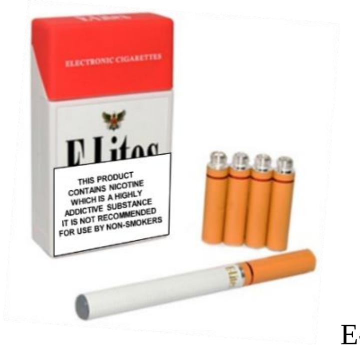

E-cigarette #1

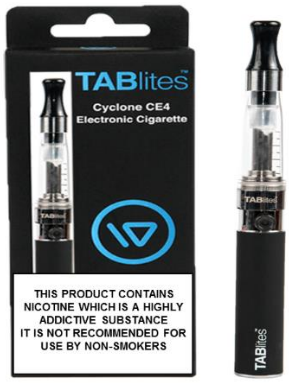

E-cigarette #2

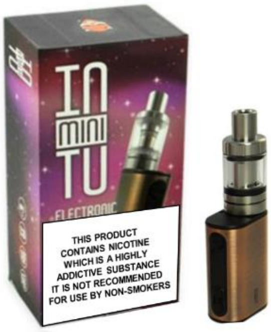

E-cigarette #3

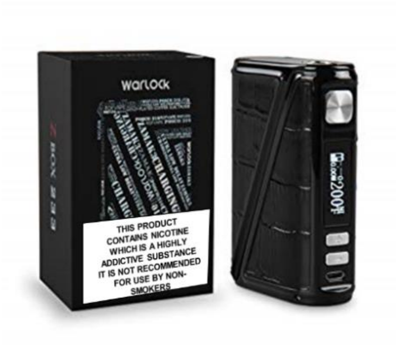

E-cigarette #4

COMP CONDITION

E-cigarette #1
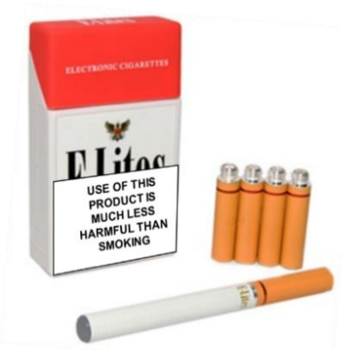

E-cigarette #2
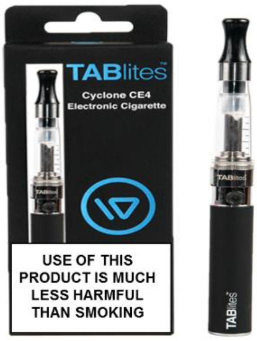

E-cigarette #3
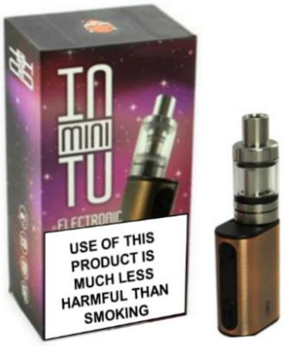

E-cigarette #4
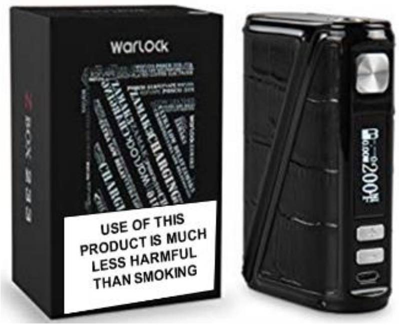

TPD1+COMP CONDITION

E-cigarette #1
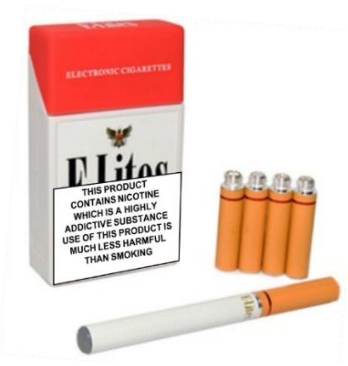

E-cigarette #2
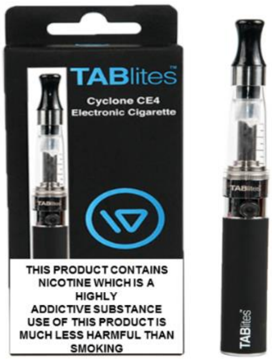

E-cigarette #3
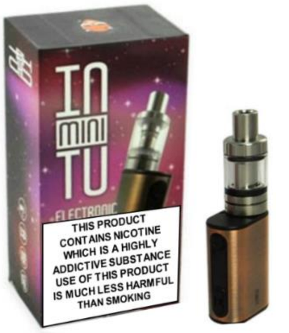

E-cigarette #4
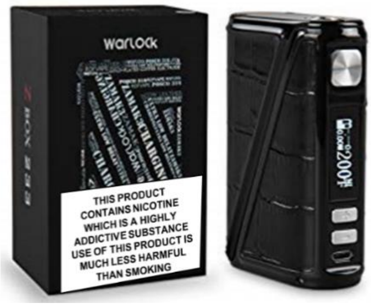

TPD2+COMP CONDITION

E-cigarette #1

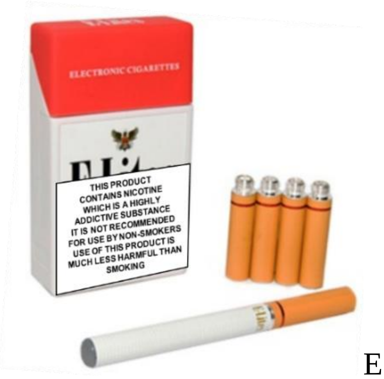

E-cigarette #2

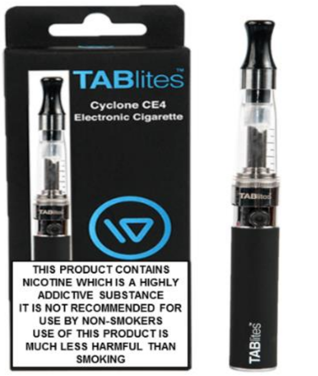

E-cigarette #3

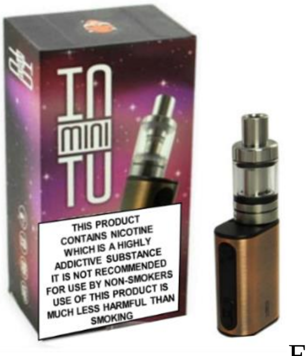

E-cigarette #4

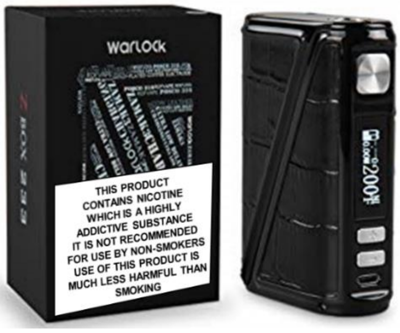

NO MESSAGE CONDITION

E-cigarette #1

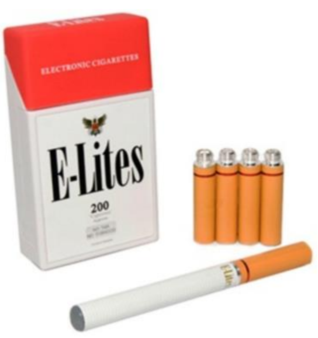

E-cigarette #2

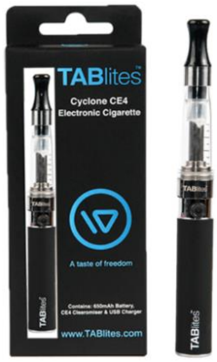

E-cigarette #3 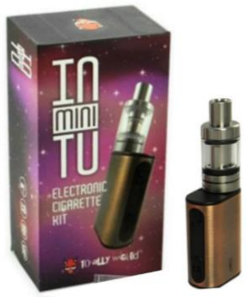

E-cigarette #4 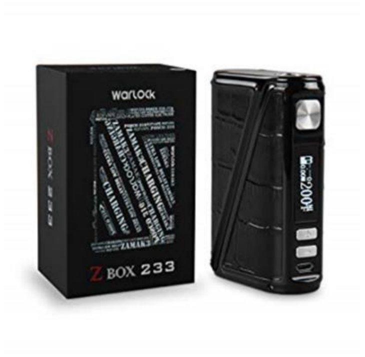

Figure 1. Randomisation of stimuli per condition for each message on EC packs
