# Supplementary material for: Communicating the relative health risks of E-cigarettes: An online experimental study exploring the effects of a comparative health message versus the EU nicotine addiction warnings on smokers’ and non-smokers’ risk perceptions and behavioural intentions
Source: Addict Behav. 2020 Feb;101:106177. doi: 10.1016/j.addbeh.2019.106177 (PMC6891257; doi:10.1016/j.addbeh.2019.106177)
Supplement: Supplementary data 2 [file mmc2.doc]

| Table 2. Demographics by Conditions (N = 2495) | |  |  |  |  |  |  |  |  | |  |
| --- | --- | --- | --- | --- | --- | --- | --- | --- | --- | --- | --- |
| **Message conditions** | **TPD1** | | **TPD2** | **COMP** | **TPD1+^a^** | **TPD2+^a^** | **No message** |  |  | |  |
| **Demographics** | **n** | | **n** | **n** | **n** | **n** | **n** | **χ^2^ (p)** |  | |  |
| **Sex** | **-** | | **-** | **-** | **-** | **-** | **-** | 1.66(.89)^b^ |  | |  |
| Male | 199 | | 199 | 182 | 198 | 207 | 188 |  |  | |  |
| Female | 216 | | 239 | 190 | 234 | 224 | 219 |  |  | |  |
| **Ethnicity** | **-** | | **-** | **-** | **-** | **-** | **-** | 0.36(.90)^c^ |  | |  |
| White | 377 | | 409 | 344 | 401 | 399 | 373 |  |  | |  |
| Black/African/Caribbean | 8 | | 2 | 6 | 6 | 8 | 8 |  |  | |  |
| Mixed/Multiple ethnic background | 6 | | 8 | 7 | 9 | 7 | 7 |  |  | |  |
| South Asian/Indian/Pakistani/Bangladeshi | 16 | | 11 | 11 | 12 | 12 | 13 |  |  | |  |
| Chinese/Other Asian background | 4 | | 5 | 3 | 0 | 4 | 4 |  |  | |  |
| Other | 4 | | 3 | 1 | 4 | 1 | 1 |  |  | |  |
| **Occupation** | **-** | | **-** | **-** | **-** | **-** | **-** | 18.96(.22)^b^ |  | |  |
| Routine and manual | 134 | | 115 | 121 | 127 | 117 | 109 |  |  | |  |
| Intermediate | 66 | | 78 | 76 | 76 | 96 | 85 |  |  | |  |
| Managerial & professional | 113 | | 129 | 100 | 114 | 122 | 106 |  |  | |  |
| Never worked & Long term unemployed | 102 | | 116 | 75 | 115 | 96 | 107 |  |  | |  |
| **Highest qualification to date** | **-** | | **-** | **-** | **-** | **-** | **-** | 36.54(.40)^b^ |  | |  |
| Degree (or equivalent) | 114 | | 136 | 110 | 110 | 136 | 123 |  |  | |  |
| Higher education (below degree level) | 38 | | 48 | 37 | 40 | 54 | 42 |  |  | |  |
| A-levels or Highers | 77 | | 77 | 70 | 81 | 65 | 75 |  |  | |  |
| ONC or National level BTEC | 31 | | 23 | 26 | 33 | 15 | 23 |  |  | |  |
| O-Level or GCSE equivalent (A-C) | 77 | | 63 | 54 | 76 | 74 | 67 |  |  | |  |
| GCSE (D-E), CSE (2-5) or standard grade (4-6) | 39 | | 48 | 50 | 55 | 46 | 39 |  |  | |  |
| Other qualifications | 19 | | 21 | 15 | 18 | 13 | 14 |  |  | |  |
| No formal qualifications | 20 | | 22 | 10 | 19 | 28 | 24 |  |  | |  |
| **Age segments** | **-** | | **-** | **-** | **-** | **-** | **-** | 5.51(.36)^b^ |  | |  |
| 18-49 | 223 | | 235 | 221 | 234 | 252 | 233 |  |  | |  |
| 50+ | 192 | | 203 | 151 | 198 | 179 | 174 |  |  | |  |
| *Note*. ^a^ TPD1+ and TPD2+ correspond to TPD1+COMP and TPD2+COMP respectively; ^b^ p values for each comparisons are reported using Pearson Chi Square; ^c^ Cramer’s V (p) is reported due to 12 cells of expected count of < 5. | | | | | | | | | |  | |
